# The contribution of platelets to peripheral BDNF elevation in children with autism spectrum disorder

**DOI:** 10.1038/s41598-021-97367-4

**Published:** 2021-09-13

**Authors:** Cristan A. Farmer, Audrey E. Thurm, Bianca Honnekeri, Paul Kim, Susan E. Swedo, Joan C. Han

**Affiliations:** 1 Pediatrics and Developmental Neuroscience Branch, National Institute of Mental Health, National Institutes of Health, Bethesda, MD 20892 USA; 2 Grant Government Medical College and Sir J.J. Group of Hospitals, Mumbai, 400008 India; 3 Clinical Electives Program, National Institutes of Health, Bethesda, MD 20892 USA; 4 Human Brain Collection Core, National Institute of Mental Health, National Institutes of Health, Bethesda, MD 20892 USA; 5 Unit on Metabolism and Neuroendocrinology, Eunice Kennedy Shriver National Institute of Child Health and Human Development, National Institutes of Health, Bethesda, MD 20892 USA; 6 Division of Pediatric Endocrinology and Diabetes, Department of Pediatrics, Icahn School of Medicine at Mount Sinai, New York, NY 10029 USA

**Keywords:** Autism spectrum disorders, Neurotrophic factors

## Abstract

Brain-derived neurotrophic factor (BDNF), a key peptide in neurocognitive development, has been reported to be elevated in the serum of children with autism spectrum disorder (ASD). In a few studies, however, no differences or the converse have been documented. As a secondary analysis of a natural history study, we examined differences in ELISA serum BDNF between a group of children aged 1 to 9 years (69% white) with ASD (n = 94) and those with typical development (n = 52) or non-ASD developmental delay (n = 21), while accounting for the potential confounding effects of platelet quantity. Platelet counts were measured within 4 h of blood draw using an automated cell counter. Taqman single nucleotide polymorphism (SNP) assays were used to genotype 11 SNPs within the *BDNF* locus. Unadjusted mean BDNF concentration was higher in children with ASD than in children with typical development (standardized mean difference = 0.23; 95% CI 0.07, 0.38), but not children with non-ASD developmental delay. The magnitude of this difference was reduced after adjusting for platelet count (standardized mean difference = 0.18; 95% CI 0.02, 0.33). Although some *BDNF* SNPs were related to BDNF concentration, the distributions of these genotypes did not differ across diagnostic groups. This study replicates previous work suggesting that average serum BDNF concentration is higher in ASD compared to typical development, and extends that work by highlighting the potentially confounding role of platelet counts. The etiology of platelet count differences warrants further elucidation. Nonetheless, our results suggest that elevation in BDNF may be partially explained by higher platelet counts in children with ASD, an association that should be considered in future analysis and interpretation.

**Registration:** NCT00298246

## Introduction

Brain-derived neurotrophic factor (BDNF) is the most abundant member of the neurotrophin family and plays an important role in neuronal development and survival^1^. It has key functions in synaptic plasticity, energy balance, memory, learning, and neuropsychiatric and neurodegenerative pathologies^2–5^.

Based on the premise that they may be reflective of brain BDNF levels, peripheral blood *BDNF* RNA expression^6^ and BDNF protein concentrations of individuals with autism spectrum disorder (ASD) have been investigated in multiple studies^7–12^ and predominantly are found to be increased in ASD. The largest available meta-analysis found that across 27 studies encompassing 6,571 unique participants, BDNF was elevated in the blood of children with ASD compared to typically developing children^7^; four other smaller meta-analyses have drawn the same conclusion^8–11^, as has at least one very recently published study not included in the previous meta-analyses^12^. Importantly, however, none of these studies reported controlling for the platelet count.

Peripheral BDNF is mostly stored in platelets and released upon degranulation^13^. Commercially available BDNF enzyme-linked immunosorbent assay (ELISA) kits recommend several steps to ensure accurate measurement of BDNF. These include concomitant measurement of and normalization of data to platelet count, and special collection and processing procedures that require extra aliquot centrifugation steps to ensure adequate platelet removal from plasma samples prior to platelet degranulation. Platelet count must be determined on the same day as blood collection and cannot be performed on stored frozen samples, and blood collected for clinical and research purposes typically would not undergo the additional processing steps to remove platelets prior to degranulation. Thus, in situations where neither platelet counts nor platelet-poor samples are available, the interpretation of differences in BDNF depends on the assumption that platelet counts are similar across the comparison groups being investigated. This is an especially relevant consideration for studies of BDNF in ASD, as some studies have found evidence for elevated platelet count in groups of children with ASD^14,15^ (others report no difference^16,17^, and no meta-analysis is available).

In the current study, we examined differences in serum BDNF between a group of children aged 1 to 9 years with ASD and those with typical development, while accounting for the potential confounding effects of platelet quantity. We extend the literature by including a group of children with non-ASD developmental delay and investigating the associations among diagnosis, BDNF concentrations, and common genetic variants of the *BDNF* gene while controlling for platelet count. Given that at least some data suggest differences in platelet counts between ASD, we postulate that the differences among groups in serum BDNF concentration may reflect differences in platelet counts in these pre-pubertal children.

## Methods and materials

Participants were drawn from a longitudinal natural history study (NCT00298246) at the National Institute of Mental Health, Bethesda, MD. This study was approved by the Institutional Review Board of the Neuroscience Institutes of the National Institutes of Health and was conducted in accordance with relevant guidelines and regulations. Participants were recruited using flyers and electronic message boards for service providers in the Washington, DC and Bethesda, Maryland area. Informed consent from the legal guardian (and assent from the child when applicable) was obtained for all participants. Children diagnosed with DSM-IV-TR autistic disorder were enrolled in the autism spectrum disorder (ASD) group, those with significantly impaired cognitive ability but without ASD were enrolled in the non-ASD developmental delay (DD) group, and children with no behavioral or psychological concerns were enrolled in the typical development (TYP) group. As part of the observational study, participants were assessed at 6-month or 1-year intervals. The procedures of this study are described in full elsewhere^18,19^.

Venous blood samples were obtained in the morning (most participants in the ASD group had fasted, the remaining children had not). Platelet counts in potassium EDTA tubes were measured within 4 h of blood draw in the NIH Clinical Center Laboratory using an automated cell counter. Serum separator tubes were permitted to clot at room temperature prior to centrifugation, and serum aliquots were stored at − 80 °C until analysis.

Serum BDNF concentrations in all samples were measured contemporaneously using a commercial enzyme-linked immunosorbent assay (ELISA) with intra- and inter-assay variabilities of 3.8% and 7.6%, respectively, and a minimum detection limit of 20 pg/mL (Human BDNF Quantikine ELISA, Catalog #DBD00, R&D Systems, Minneapolis, MN, USA). The average value of duplicate measurements was used for data analyses.

Genomic DNA was extracted from the buffy coat layer of venous whole blood using QIAamp DNA Blood Mini kits (Qiagen, Valencia, CA) and genotyped for 11 common single nucleotide polymorphisms (SNPs) within the loci for *BDNF* (rs1491851, rs1491850, rs908867, rs7127507, rs12291063, rs11030104, rs12273539, rs6265, rs10501087, rs925946, and rs11030096) using Taqman SNP Genotyping Assays (Applied Systems, Thermo Fisher Scientific, Waltham, MA). The *BDNF* SNPs were chosen based on coverage across the 3’ downstream, gene coding, and 5’ upstream regions of *BDNF*^20^. Most BDNF SNPs are intronic in location and those in the coding region are predominantly in the pro-domain of BDNF. Therefore, it is hypothesized that the effect of these SNPs on BDNF RNA and mature protein levels are mediated through altered transcriptional regulation via protein enhancer^21^ and micro-RNA^22^ mechanisms, splicing patterns^23^, and protein processing^24^.

The primary analysis was a general linear model predicting BDNF concentration from group (ASD, DD, or TYP). In the secondary analysis, we evaluated the impact of platelet count on the group comparison. In standard exploratory data analysis, we evaluated other demographic features as potential covariates (age, biological sex, race, and BMI), but none showed meaningful differences across groups or association with BDNF, and therefore, none were carried forward into analysis (see supplementary materials). Seven participants were missing platelet count data; for the purpose of comparison, the primary model was also run in complete cases. Model assumptions were evaluated via visual inspection of plotted residuals. As a result, two adjustments were made: BDNF values were square-root transformed and residual variance was estimated separately by diagnostic group.

The planned analysis for genotype distribution was ordinal logistic regression, predicting the probability of the minor variant (versus heterozygous or major variants). This model requires sufficient cell size for each group x genotype comparison; we set this threshold at n = 10. Within the DD group, there was cell size < 10 even after collapsing the heterozygous and minor variants of all SNPs. Thus, although descriptive data are provided for the DD group, they were not included in parametric analysis. For two SNPs (RS908867 and RS12273539), cell size for the TYP group remained < 10 after collapsing heterozygous and minor variants, so parametric analysis was not performed. RS1491851 was analyzed without collapsing variants (the planned ordinal logistic regression), and the remaining eight SNPs were analyzed using binary logistic regression (major versus combined heterozygous and minor; RS1491850, RS7127507, RS12291063, RS11030104, RS6265, RS10501087, RS925946, RS11030096). Finally, the relationship between genotype and BDNF concentration was assessed using the generalized linear model, with genotype as the predictor and platelet count as covariate. Because these analyses utilized the entire sample, it was necessary to collapse the minor variant into the heterozygous variant for only four SNPs (RS11030104, RS12273539, RS6265, and RS10501087). For these analyses, genotype was treated as a linear predictor with heterogeneous variance.

Because these were secondary analyses within a larger study, no a priori power calculation was performed. Consistent with the current recommendations of the American Statistical Association^25^, specific uncorrected p-values are provided, and parameter estimates and their 95% confidence intervals are interpreted rather than employing a threshold for “statistical significance.” Where applicable, standardized mean differences (SMD) were calculated as the difference in means divided by the pooled standard deviation. SAS/STAT 9.4 was used for these analyses (model syntax appears in supplementary materials).

### Ethics approval and consent to participate

A legal guardian provided written consent for participation in this protocol, approved by the NIH Combined Neurosciences Institutional Review Board.

## Results

**Figure 1:**
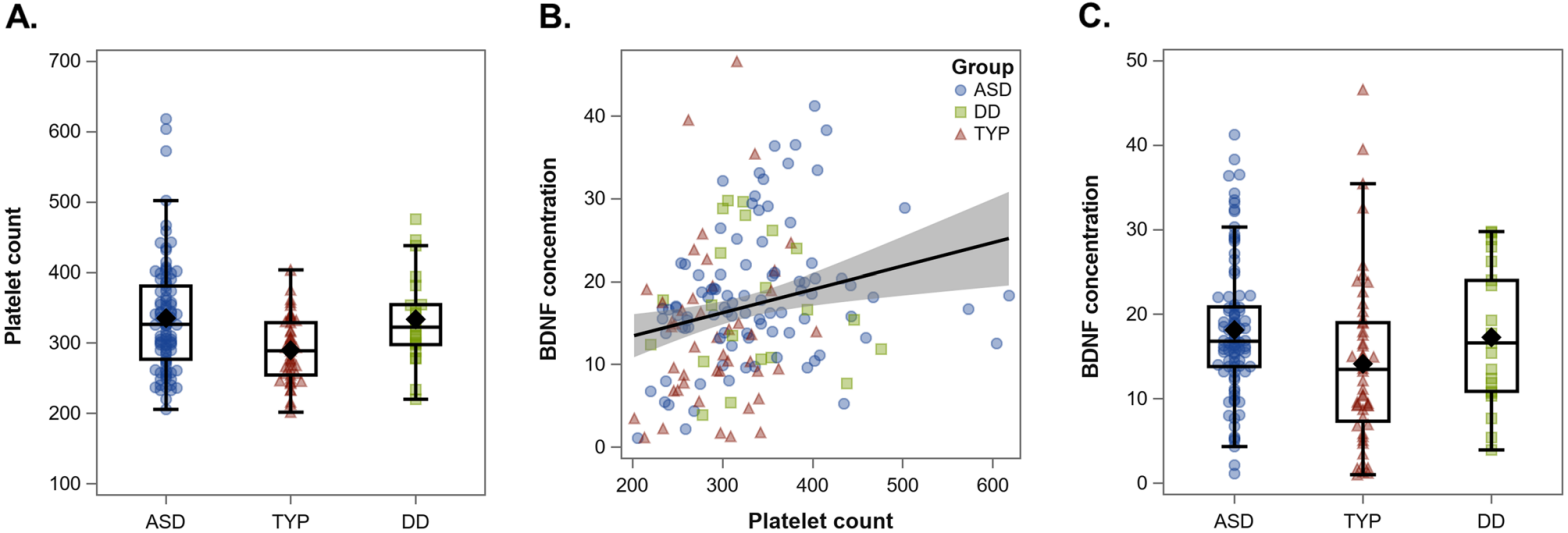
Plots of raw data. *ASD* autism spectrum disorder, *TYP* typical development, *DD* developmental delay. Panel (**A**) shows platelet count by group. Test statistics for pairwise comparisons were as follows: ASD:TYP, t(157) = 4.17, p < 0.0001; ASD:DD, t(157) = 0.06, p = 0.096; DD:TYP, t(157) = 2.75, p = 0.007. Panel (**B**) shows the correlation between platelet count and BDNF concentration (ng/mL) (r = 0.23). This correlation increased after excluding extreme values of platelet count and/or BDNF concentration (ng/mL). Panel (**C**) shows BDNF concentration (ng/mL) by group. See Table 2 for associated inferential statistics.

The sample for this analysis comprised 94 children in the ASD group, 21 children in the DD group, and 52 children in the TYP group (age range 1.25 to 9.21 years). All participants, both male and female, were Tanner stage 1 and prepubertal. Participants are descriptively summarized in Table 1. Through standard exploratory data analysis (Supplementary Table 1), we observed that platelet count in the ASD (SMD = 0.33, 95% CI [0.18, 0.49]) and DD (SMD = 0.22 [0.06, 0.38]) groups were higher than in the TYP group (ASD and DD did not differ, SMD = 0.004 [− 0.15, + 0.16]) (see Fig. 1A). Using age- and sex-based reference ranges supplied by our clinical laboratory, 19% of ASD, 14% of DD, and none of TYP had elevated platelet counts. Platelet count was moderately and positively correlated with BDNF (*r* = 0.23; see Fig. 1B); this was true even with participants with extreme values in platelet count and/or BDNF concentration were excluded.

**Table 1:** Descriptive data.

	ASD (n = 94)		DD (n = 21)		TYP (n = 52)	
	*n*	%	*n*	%	*n*	%
Male	76	81	13	62	37	71
White	66	70	12	57	38	73
Anticonvulsant medication	9	10	3	14	0	
Antipsychotic medication	8	9	1	5	0	
Selective serotonin reuptake inhibitor	2	2	0		0	

	*M*	*SD*	*M*	*SD*	*M*	*SD*
Age (years)	4.91	1.59	4.91	1.49	4.27	1.65
Vineland-II composite (standard score)	64.90	8.79	71.90	9.34	103.54	9.26
Nonverbal developmental quotient	56.02	18.75	57.51	17.06	111.59	14.01
BMI Z-score	0.52	1.21	0.02	1.28	0.51	1.09
Platelet count (10^3^/mm^3^)	336	79	334	66	290	46
BDNF (ng/mL)	18.14	8.16	17.28	8.19	14.18	9.84

Some data were missing: BMI, n = 9 in ASD, n = 3 in DD, n = 3 in TYP. Platelets, n = 7 in TYP. Psychotropic medications, n = 3 TYP. Eighteen (19%) children in the ASD group, three (14%) in the DD group, and none of the participants in the TYP group had elevated platelets, according to age- and sex-based clinical laboratory reference ranges. Age ranges in the groups were ASD 2.01–9.21 years, DD 2.83–8.07 years, and TYP 1.25 to 7.86 years.

Descriptively, the ASD group had the highest BDNF concentrations (see Table 1 and Fig. 1C). The results of the primary and secondary models are summarized in Table 2 (full results are shown in Supplementary Table 2). BDNF concentration was higher in ASD than in TYP (SMD = 0.23 [0.07, 0.38]), and slightly higher in DD than in TYP (SMD = 0.14, [− 0.02, 0.29]), but ASD and DD did not differ from one another. In the secondary analysis, platelet count partially accounted for the difference in BDNF between ASD and TYP, reducing the magnitude of their standardized difference by about 20% (SMD_adj_ = 0.18 [0.02, 0.33]).

**Table 2:** Results of primary and secondary models comparing BDNF concentration (square-root transformed) among study groups.

	ASD versus TYP	DD versus TYP	ASD versus DD
**Model 1: no covariate**			
Test statistic	t(164) = 2.91, p = 0.004	t(164) = 1.73, p = 0.09	t(164) = 0.44, p = 0.66
SMD [95% CI]	0.23 [0.07, 0.38]	0.14 [− 0.02, 0.29]	0.03 [− 0.12, 0.19]
**Model 2: platelet covariate**			
Test statistic	t(156) = 2.19, p = 0.03	t(156) = 1.25, p = 0.21	t(156) = 0.41, p = 0.68
SMD [95% CI]	0.18 [0.02, 0.33]	0.10 [− 0.06, 0.26]	0.03 [− 0.13, 0.19]

*SMD* standardized mean difference, *CI* confidence interval, *ASD* autism spectrum disorder (n = 94), *TYP* typical development (n = 52), *DD* developmental delay (n = 21). Outcome was square-root transformed BDNF (pg/mL). SMD was calculated using estimated mean difference. Model 1 was also performed with complete cases (i.e., excluding the seven TYP participants without platelet count); effect size estimates were identical to those shown in this table (see Supplementary Materials). Full results for all models are shown in the Supplementary Materials.

Frequency counts for each genotype are shown in Supplementary Table 3a. As described in the Methods, the DD group was excluded from parametric comparison of genotype distributions because of small cell size, and for the comparison of ASD and TYP, the minor variants were collapsed with the heterozygous variants for some SNPs (see summary in Supplementary Table 3b). There was no difference in the odds of the minor allele (or combined heterozygous and minor alleles) between ASD and TYP for any SNP (see Supplementary Table 3c).

In the full sample, four SNPs were associated with BDNF concentration (see Supplementary Table 4b). Each additional minor allele was associated with lower BDNF concentration for RS11030096 [t(153) = − 2.23, p = 0.03] and RS12291063 [t(153) = − 2.21, p = 0.03], while each additional minor allele was associated with higher BDNF concentration for RS12273539 [t(156) = 2.09, p = 0.04] and RS908867 [t(157) = 2.07, p = 0.04].

## Discussion

This study replicates prior reports of higher serum BDNF concentrations in children with ASD compared to typically developing children. Previous studies that assessed BDNF in ASD did not control for platelet count, which is a potential confounder of the measurement of BDNF. We extended the literature by documenting that in our study, differences in platelet count accounted for about 20% of the difference in BDNF between ASD and TYP. We did not find evidence that genotype of any of the 11 SNPs within the loci for *BDNF*, the distributions of which were very similar between ASD and TYP, explained this difference in BDNF concentration.

The difference in BDNF concentration between ASD and TYP groups in this study was small; average raw values differed by about 4 ng/mL (SMD = 0.23), and after adjusting for platelets, the estimated difference was about 3 ng/mL (SMD = 0.18). Although the weight of the distribution was slightly shifted in the ASD group relative to the TYP group, the distributions of raw BDNF values between groups were completely overlapping. Still, the difference observed in this study is consistent with previous literature^7–11^, and our estimate of effect size is on the low end of the range estimated by the largest available meta-analysis^7^. Thus, although our findings corroborate those available from other studies in supporting the existence of a difference in BDNF concentration between ASD and BDNF, they also suggest that studies may obtain a more precise (and likely smaller) estimate of the true difference by accounting for the effect of platelet count on BDNF concentration.

Some existing research supports the idea that a true population-level difference between ASD and typically developing children in platelet count may exist, although the explanation for increased platelet counts in ASD (and DD) is unclear. One hypothesis arises from observations that the signaling pathway associated with mammalian target of rapamycin (mTOR) is upregulated in both idiopathic and syndromic ASD^26–30^ and that mTOR activation increases proliferation of platelet precursor megakaryocytes^31^.

Because BDNF appears to have a role in preventing pathologic clot formation through reduction in fibrin formation, the possibility remains that the higher BDNF observed in ASD may be causing altered clotting dynamics although this has not been reported as clinically significant in the extant literature and remains to be explored^24^. The lack of association of *BDNF* SNPs with ASD diagnosis in our cohort partially corroborates a recent case–control study^25^ that showed no significant associations of *BDNF* variants and ASD diagnosis or BDNF concentrations in dried neonatal cord blood spots. Together these findings suggest that other determinants of BDNF concentration besides underlying genetic predisposition may be important to consider.

## Limitations

The results of this study must be interpreted in the context of several limitations. The group sizes are small, which means that we were powered only to detect relatively large differences among groups. The sample was mostly white, which may reflect bias in ascertainment and may affect the extent to which these results generalize to the broader population. The ASD group was the only one which had fasted prior to the blood draw. Because platelets are an acute phase reactant and could vary based on illness or other exogenous conditions, the lack of repeated measurement is a limitation. However, given that all participants reported being subjectively clinically well at the time of blood draw, variability due to acute illness would be expected to be similar across groups. The cross-sectional data also prevent the attribution of causality for the associations observed in this study.

## Conclusion

People with autism benefit from studies investigating the pathophysiology of autism because interventions may be designed to address potential contributors to autism symptom severity. Our work supports previous studies in documenting elevated BDNF in ASD relative to typically developing children, but highlights the fact that the magnitude of this difference may be affected by platelet count, which has historically been ignored in studies of ASD. We therefore recommend that future studies examining the association between ASD and BDNF concentrations in peripheral circulation should account for differences in platelet count as a potential confounder.

## Supplementary Information

Supplementary Information.

## Data Availability

The datasets analyzed during the current study are available from the corresponding author (JCH) on reasonable request.
