# Supplementary material for: The contribution of platelets to peripheral BDNF elevation in children with autism spectrum disorder
Source: Sci Rep. 2021 Sep 13;11:18158. doi: 10.1038/s41598-021-97367-4 (PMC8438074; doi:10.1038/s41598-021-97367-4)
Supplement: Supplementary file 1 — Supplementary Information. [file 41598_2021_97367_MOESM1_ESM.docx]

**SUPPLEMENTARY MATERIALS**

**The contribution of platelets to p****eripheral BDNF elevation in children with autism spectrum disorder**

Cristan A. Farmer, Ph.D.^1^, Audrey E. Thurm, Ph.D. ^1^, Bianca Honnekeri, MBBS^2,3^, Paul Kim, M.D., Ph.D.^4^, Susan E. Swedo, M.D.^1^, Joan C. Han, M.D.*^1,5,6^

Cristan A. Farmer, Ph.D.

farmerca@mail.nih.gov

^1^Pediatrics and Developmental Neuroscience Branch, National Institute of Mental Health, National Institutes of Health, Bethesda, MD 20892

Audrey E. Thurm, Ph.D.

athurm@mail.nih.gov

^1^Pediatrics and Developmental Neuroscience Branch, National Institute of Mental Health, National Institutes of Health, Bethesda, MD 20892

Bianca Honnekeri, MBBS

biancahonnekeri@gmail.com

^2^Grant Government Medical College and Sir J.J. Group of Hospitals, Mumbai 400008, India

^3^Clinical Electives Program, National Institutes of Health, Bethesda, MD 20892

Paul Kim, M.D., Ph.D.

paul.kim@nih.gov

^4^Human Brain Collection Core, National Institute of Mental Health, National Institutes of Health, Bethesda, MD 20892

Susan E. Swedo, M.D.

swedos@mail.nih.gov

^1^Pediatrics and Developmental Neuroscience Branch, National Institute of Mental Health, National Institutes of Health, Bethesda, MD 20892

*Joan C. Han, M.D.

joan.han@mssm.edu

^1^Pediatrics and Developmental Neuroscience Branch, National Institute of Mental Health, National Institutes of Health, Bethesda, MD 20892

^5^Unit on Metabolism and Neuroendocrinology, Eunice Kennedy Shriver National Institute of Child Health and Human Development, National Institutes of Health, Bethesda, MD 20892

^6^Division of Pediatric Endocrinology and Diabetes, Department of Pediatrics, Icahn School of Medicine at Mount Sinai, New York, NY 10029

**Contents**

1. [Exploratory Data Analysis](#EDA)
   1. [Descriptives](#EDAdescriptive)
   2. [Group differences in platelet count](#EDAplatelet)
   3. [Correlation of platelet and demographic variables with BDNF](#EDAcorrelation)
2. [Results of Primary and Secondary Analyses](#PrimaryResults)
   1. [Comparison of BDNF by diagnostic group](#PrimaryResultsNoCovar)
   2. [Comparison of BDNF by diagnostic group, controlling for platelet count](#SecondaryResultsCovar)
   3. [Comparison of BDNF by diagnostic group, excluding participants without platelet count](#PrimaryResultsSubset)
3. [Results of Genotype Analyses](#GenotypeResults)
   1. [Genotype distributions by group](#GenotypeFrequencies)
   2. [Comparison of genotype distributions by group](#GenotypeFreqsTests)
4. [Results of Genotype and BDNF Analyses](#GenotypeBDNFResults)
   1. [Comparison of BDNF by genotype](#GenotypeBDNFResultsModels)
   2. [Summary of the effect of genotype on estimated square-root transformed BDNF concentration (summary)](#GenotypeBDNFLSMeans)
5. **Exploratory Data Analysis.** Summary: No demographic variables (only platelet count) were included as covariates in the comparison of BDNF amongst groups.
   1. Descriptive statistics: BDNF, platelet count, and demographic variables

| **Variables** | **N** | **Mean** | **Std Dev** | **Sum** | **Minimum** | **Maximum** |
| --- | --- | --- | --- | --- | --- | --- |
| BDNF (ng/mL) | 167 | 16.79976 | 8.85363 | 2806 | 1.00788 | 46.62526 |
| Platelet Count (10^3^/mm^3^) | 160 | 322.65625 | 72.33074 | 51625 | 202.00000 | 618.00000 |
| Age (years) | 167 | 4.70654 | 1.61092 | 785.99274 | 1.25479 | 9.20548 |
| BMI Z-score | 152 | 0.45864 | 1.18313 | 69.71315 | -3.13139 | 2.80581 |
| Sex (Male/Female) | 167 | 1.24551 | 0.43168 | 208.00000 | 1.00000 | 2.00000 |
| Race (White/Non-White) | 167 | 1.32934 | 0.47139 | 222.00000 | 1.00000 | 2.00000 |

- 1. Generalized linear model to compare platelet count (square-root transformed) among groups.

| **Type 3 Tests of Fixed Effects** | | | | |
| --- | --- | --- | --- | --- |
| **Effect** | **Num DF** | **Den DF** | **F Value** | **Pr > F** |
| **Diagnosis_CaT3** | 2 | 157 | 9.82 | <.0001 |

| **Least Square Means** | | | | | | | | |
| --- | --- | --- | --- | --- | --- | --- | --- | --- |
| **Diagnosis** | **Estimate** | **Standard Error** | **DF** | **t Value** | **Pr > \|t\|** | **Alpha** | **Lower** | **Upper** |
| **Autism** | 18.2082 | 0.2142 | 157 | 84.99 | <.0001 | 0.05 | 17.7851 | 18.6314 |
| **Developmental Delay** | 18.1836 | 0.3887 | 157 | 46.78 | <.0001 | 0.05 | 17.4159 | 18.9514 |
| **Typical** | 16.9774 | 0.2030 | 157 | 83.65 | <.0001 | 0.05 | 16.5765 | 17.3783 |

| **Differences of Least Square Means** | | | | | | | | | |
| --- | --- | --- | --- | --- | --- | --- | --- | --- | --- |
| **Diagnosis** | **Diagnosis** | **Estimate** | **Standard Error** | **DF** | **t Value** | **Pr > \|t\|** | **Alpha** | **Lower** | **Upper** |
| **Autism** | **Developmental Delay** | 0.02460 | 0.4438 | 157 | 0.06 | 0.9559 | 0.05 | -0.8521 | 0.9013 |
| **Autism** | **Typical** | 1.2309 | 0.2951 | 157 | 4.17 | <.0001 | 0.05 | 0.6479 | 1.8138 |
| **Developmental Delay** | **Typical** | 1.2062 | 0.4385 | 157 | 2.75 | 0.0066 | 0.05 | 0.3401 | 2.0724 |

- 1. Pearson correlations with BDNF to evaluate need for covariation (point-biserial for categorical).

|  |  | **Platelet (square-root transformed)** | **Age** | **BMI** | **Sex** | **Race** |
| --- | --- | --- | --- | --- | --- | --- |
| \| **BDNF (square-root transformed)** \| \| --- \| \|  \| | \| r \| \| --- \| \| p \| \| n \| | \| 0.27372 \| \| --- \| \| 0.0005 \| \| 160 \| | \| 0.07696 \| \| --- \| \| 0.3229 \| \| 167 \| | \| 0.07320 \| \| --- \| \| 0.3701 \| \| 152 \| | \| 0.01473 \| \| --- \| \| 0.8502 \| \| 167 \| | \| 0.05731 \| \| --- \| \| 0.4619 \| \| 167 \| |

1. **Results of primary and secondary analysis:** Full model results for (a) primary model with no covariate, (b) secondary model controlling for platelet count, and (c) primary model run in subset of participants with valid platelet count (i.e., excluding n=7 TYP). Note that standardized mean differences were calculated as the estimated difference divided by the standard error of that difference, which was then divided by the square root of the degrees of freedom for the comparison.
   1. **Primary model with no covariates**

proc mixed data=data plots=all;

class group;

model bdnf_sqrt = group;

repeated / group= group ;

lsmeans group / pdiff cl;

run;

| **Model Information** | |
| --- | --- |
| **Data Set** | WORK.BDNF |
| **Dependent Variable** | bdnf_sqrt |
| **Covariance Structure** | Variance Components |
| **Group Effect** | group |
| **Estimation Method** | REML |
| **Residual Variance Method** | None |
| **Fixed Effects SE Method** | Model-Based |
| **Degrees of Freedom Method** | Between-Within |

| **Class Level Information** | | |
| --- | --- | --- |
| **Class** | **Levels** | **Values** |
| group | 3 | Autism Developmental Delay Typical |

| **Dimensions** | |
| --- | --- |
| **Covariance Parameters** | 3 |
| **Columns in X** | 4 |
| **Columns in Z** | 0 |
| **Subjects** | 167 |
| **Max Obs per Subject** | 1 |

| **Number of Observations** | |
| --- | --- |
| **Number of Observations Read** | 167 |
| **Number of Observations Used** | 167 |
| **Number of Observations Not Used** | 0 |

| **Iteration History** | | | |
| --- | --- | --- | --- |
| **Iteration** | **Evaluations** | **-2 Res Log Like** | **Criterion** |
| **0** | 1 | 512.69293277 |  |
| **1** | 1 | 506.82108536 | 0.00000000 |

| Convergence criteria met. |
| --- |

| **Covariance Parameter Estimates** | | |
| --- | --- | --- |
| **Cov Parm** | **Group** | **Estimate** |
| **Residual** | group **Autism** | 0.9952 |
| **Residual** | group **Developmental Delay** | 1.0711 |
| **Residual** | group **Typical** | 1.7640 |

| **Fit Statistics** | |
| --- | --- |
| **-2 Res Log Likelihood** | 506.8 |
| **AIC (Smaller is Better)** | 512.8 |
| **AICC (Smaller is Better)** | 513.0 |
| **BIC (Smaller is Better)** | 522.2 |

| **Null Model Likelihood Ratio Test** | | |
| --- | --- | --- |
| **DF** | **Chi-Square** | **Pr > ChiSq** |
| 2 | 5.87 | 0.0531 |

| **Type 3 Tests of Fixed Effects** | | | | |
| --- | --- | --- | --- | --- |
| **Effect** | **Num DF** | **Den DF** | **F Value** | **Pr > F** |
| group | 2 | 164 | 4.25 | 0.0158 |

| **Least Squares Means** | | | | | | | | | |
| --- | --- | --- | --- | --- | --- | --- | --- | --- | --- |
| **Effect** | **Diagnosis_CaT3** | **Estimate** | **Standard Error** | **DF** | **t Value** | **Pr > \|t\|** | **Alpha** | **Lower** | **Upper** |
| group | **Autism** | 4.1424 | 0.1029 | 164 | 40.26 | <.0001 | 0.05 | 3.9392 | 4.3455 |
| group | **Developmental Delay** | 4.0324 | 0.2258 | 164 | 17.85 | <.0001 | 0.05 | 3.5864 | 4.4783 |
| group | **Typical** | 3.5279 | 0.1842 | 164 | 19.15 | <.0001 | 0.05 | 3.1642 | 3.8916 |

| **Differences of Least Squares Means** | | | | | | | | | | |
| --- | --- | --- | --- | --- | --- | --- | --- | --- | --- | --- |
| **Effect** | **Diagnosis_CaT3** | **Diagnosis_CaT3** | **Estimate** | **Standard Error** | **DF** | **t Value** | **Pr > \|t\|** | **Alpha** | **Lower** | **Upper** |
| group | **Autism** | **Developmental Delay** | 0.1100 | 0.2482 | 164 | 0.44 | 0.6582 | 0.05 | -0.3800 | 0.6000 |
| group | **Autism** | **Typical** | 0.6145 | 0.2110 | 164 | 2.91 | 0.0041 | 0.05 | 0.1979 | 1.0310 |
| group | **Developmental Delay** | **Typical** | 0.5045 | 0.2914 | 164 | 1.73 | 0.0853 | 0.05 | -0.07095 | 1.0799 |


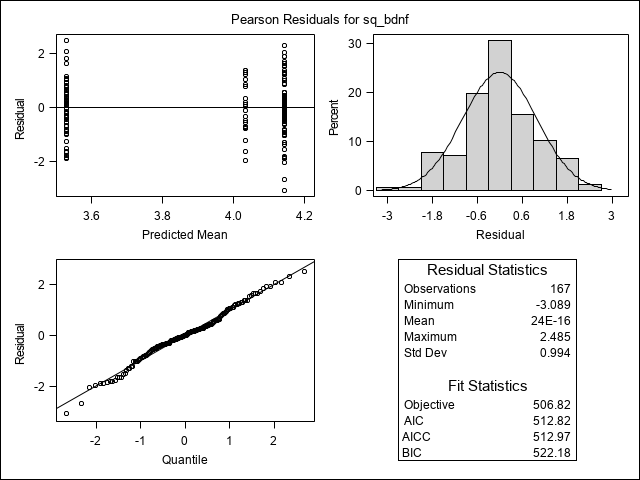

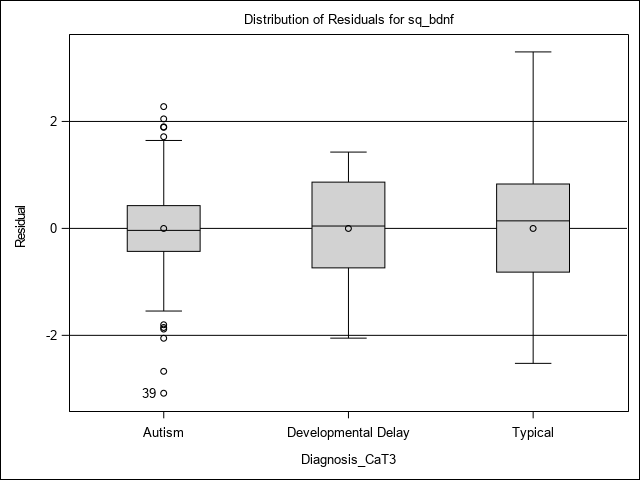


- 1. **Secondary model, controlling for platelet count**

PROC MIXED DATA=data PLOTS=all;

CLASS group;

MODEL bdnf_sqrt = platelet group;

REPEATED / GROUP= group ;

LSMEANS group / PDIFF CL;

RUN;

| **Model Information** | |
| --- | --- |
| **Data Set** | WORK.BDNF1 |
| **Dependent Variable** | Bdnf_sqrt |
| **Covariance Structure** | Variance Components |
| **Group Effect** | group |
| **Estimation Method** | REML |
| **Residual Variance Method** | None |
| **Fixed Effects SE Method** | Model-Based |
| **Degrees of Freedom Method** | Between-Within |

| **Class Level Information** | | |
| --- | --- | --- |
| **Class** | **Levels** | **Values** |
| group | 3 | Autism Developmental Delay Typical |

| **Dimensions** | |
| --- | --- |
| **Covariance Parameters** | 3 |
| **Columns in X** | 5 |
| **Columns in Z** | 0 |
| **Subjects** | 167 |
| **Max Obs per Subject** | 1 |

| **Number of Observations** | |
| --- | --- |
| **Number of Observations Read** | 167 |
| **Number of Observations Used** | 160 |
| **Number of Observations Not Used** | 7 |

| **Iteration History** | | | |
| --- | --- | --- | --- |
| **Iteration** | **Evaluations** | **-2 Res Log Like** | **Criterion** |
| **0** | 1 | 490.52356681 |  |
| **1** | 2 | 485.31016136 | 0.00000000 |

| Convergence criteria met. |
| --- |

| **Covariance Parameter Estimates** | | |
| --- | --- | --- |
| **Cov Parm** | **Group** | **Estimate** |
| **Residual** | group **Autism** | 0.9278 |
| **Residual** | group **Developmental Delay** | 1.1413 |
| **Residual** | group **Typical** | 1.6484 |

| **Fit Statistics** | |
| --- | --- |
| **-2 Res Log Likelihood** | 485.3 |
| **AIC (Smaller is Better)** | 491.3 |
| **AICC (Smaller is Better)** | 491.5 |
| **BIC (Smaller is Better)** | 500.7 |

| **Null Model Likelihood Ratio Test** | | |
| --- | --- | --- |
| **DF** | **Chi-Square** | **Pr > ChiSq** |
| 2 | 5.21 | 0.0738 |

| **Type 3 Tests of Fixed Effects** | | | | |
| --- | --- | --- | --- | --- |
| **Effect** | **Num DF** | **Den DF** | **F Value** | **Pr > F** |
| **Platelet** | 1 | 156 | 7.44 | 0.0071 |
| group | 2 | 156 | 2.39 | 0.0950 |

| **Least Squares Means** | | | | | | | | | |
| --- | --- | --- | --- | --- | --- | --- | --- | --- | --- |
| **Effect** | **Diagnosis_CaT3** | **Estimate** | **Standard Error** | **DF** | **t Value** | **Pr > \|t\|** | **Alpha** | **Lower** | **Upper** |
| group | **Autism** | 4.1013 | 0.1005 | 156 | 40.82 | <.0001 | 0.05 | 3.9028 | 4.2998 |
| group | **Developmental Delay** | 3.9980 | 0.2335 | 156 | 17.12 | <.0001 | 0.05 | 3.5368 | 4.4591 |
| group | **Typical** | 3.6162 | 0.1950 | 156 | 18.54 | <.0001 | 0.05 | 3.2310 | 4.0014 |

| **Differences of Least Squares Means** | | | | | | | | | | |
| --- | --- | --- | --- | --- | --- | --- | --- | --- | --- | --- |
| **Effect** | **Diagnosis_CaT3** | **Diagnosis_CaT3** | **Estimate** | **Standard Error** | **DF** | **t Value** | **Pr > \|t\|** | **Alpha** | **Lower** | **Upper** |
| group | **Autism** | **Developmental Delay** | 0.1033 | 0.2534 | 156 | 0.41 | 0.6841 | 0.05 | -0.3973 | 0.6039 |
| group | **Autism** | **Typical** | 0.4851 | 0.2219 | 156 | 2.19 | 0.0303 | 0.05 | 0.04674 | 0.9234 |
| group | **Developmental Delay** | **Typical** | 0.3818 | 0.3057 | 156 | 1.25 | 0.2136 | 0.05 | -0.2221 | 0.9857 |


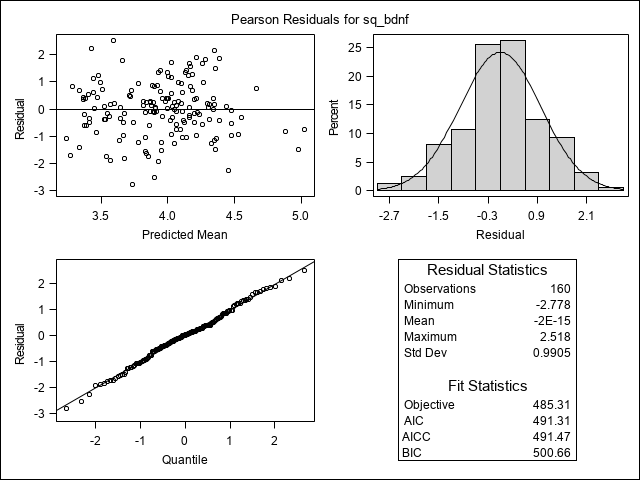

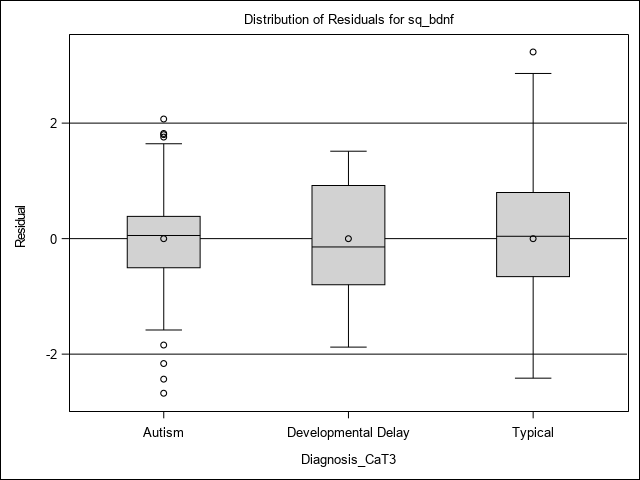


- 1. **Primary model, complete cases only (excluding n=7 TYP with no platelet count)**

proc mixed data=data plots=all;

class group;

WHERE platelet ne . ;

model bdnf_sqrt = group;

repeated / group= group ;

lsmeans group / pdiff cl;

run;

| **Model Information** | |
| --- | --- |
| **Data Set** | WORK.BDNF1 |
| **Dependent Variable** | Bdnf_sqrt |
| **Covariance Structure** | Variance Components |
| **Group Effect** | group |
| **Estimation Method** | REML |
| **Residual Variance Method** | None |
| **Fixed Effects SE Method** | Model-Based |
| **Degrees of Freedom Method** | Between-Within |

| **Class Level Information** | | |
| --- | --- | --- |
| **Class** | **Levels** | **Values** |
| **Group** | 3 | Autism Developmental Delay Typical |

| **Dimensions** | |
| --- | --- |
| **Covariance Parameters** | 3 |
| **Columns in X** | 4 |
| **Columns in Z** | 0 |
| **Subjects** | 160 |
| **Max Obs per Subject** | 1 |

| **Number of Observations** | |
| --- | --- |
| **Number of Observations Read** | 160 |
| **Number of Observations Used** | 160 |
| **Number of Observations Not Used** | 0 |

| **Iteration History** | | | |
| --- | --- | --- | --- |
| **Iteration** | **Evaluations** | **-2 Res Log Like** | **Criterion** |
| **0** | 1 | 485.24172708 |  |
| **1** | 1 | 480.76270121 | 0.00000000 |

| Convergence criteria met. |
| --- |

| **Covariance Parameter Estimates** | | |
| --- | --- | --- |
| **Cov Parm** | **Group** | **Estimate** |
| **Residual** | **Group Autism** | 0.9952 |
| **Residual** | **Group Developmental Delay** | 1.0711 |
| **Residual** | **Group Typical** | 1.6827 |

| **Fit Statistics** | |
| --- | --- |
| **-2 Res Log Likelihood** | 480.8 |
| **AIC (Smaller is Better)** | 486.8 |
| **AICC (Smaller is Better)** | 486.9 |
| **BIC (Smaller is Better)** | 496.0 |

| **Null Model Likelihood Ratio Test** | | |
| --- | --- | --- |
| **DF** | **Chi-Square** | **Pr > ChiSq** |
| 2 | 4.48 | 0.1065 |

| **Type 3 Tests of Fixed Effects** | | | | |
| --- | --- | --- | --- | --- |
| **Effect** | **Num DF** | **Den DF** | **F Value** | **Pr > F** |
| **Group** | 2 | 157 | 4.12 | 0.0181 |

| **Least Squares Means** | | | | | | | | | |
| --- | --- | --- | --- | --- | --- | --- | --- | --- | --- |
| **Effect** | **Group** | **Estimate** | **Standard Error** | **DF** | **t Value** | **Pr > \|t\|** | **Alpha** | **Lower** | **Upper** |
| **Group** | **Autism** | 4.1424 | 0.1029 | 157 | 40.26 | <.0001 | 0.05 | 3.9391 | 4.3456 |
| **Group** | **Developmental Delay** | 4.0324 | 0.2258 | 157 | 17.85 | <.0001 | 0.05 | 3.5863 | 4.4785 |
| **Group** | **Typical** | 3.5143 | 0.1934 | 157 | 18.17 | <.0001 | 0.05 | 3.1324 | 3.8963 |

| **Differences of Least Squares Means** | | | | | | | | | | |
| --- | --- | --- | --- | --- | --- | --- | --- | --- | --- | --- |
| **Effect** | **Group** | **Group** | **Estimate** | **Standard Error** | **DF** | **t Value** | **Pr > \|t\|** | **Alpha** | **Lower** | **Upper** |
| **Group** | **Autism** | **Developmental Delay** | 0.1100 | 0.2482 | 157 | 0.44 | 0.6582 | 0.05 | -0.3802 | 0.6002 |
| **Group** | **Autism** | **Typical** | 0.6281 | 0.2190 | 157 | 2.87 | 0.0047 | 0.05 | 0.1954 | 1.0607 |
| **Group** | **Developmental Delay** | **Typical** | 0.5181 | 0.2973 | 157 | 1.74 | 0.0834 | 0.05 | -0.06920 | 1.1053 |


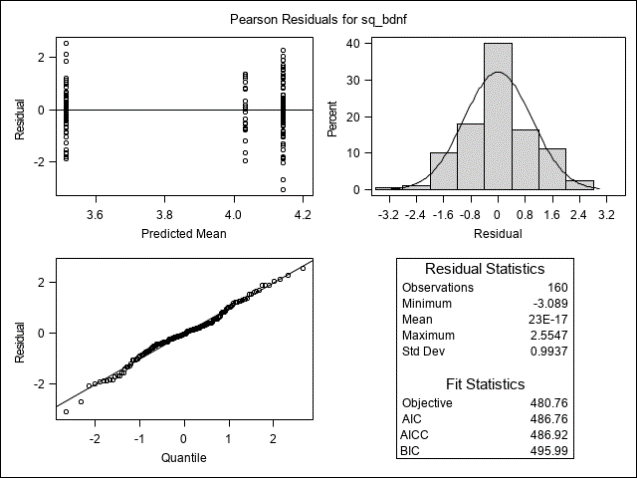

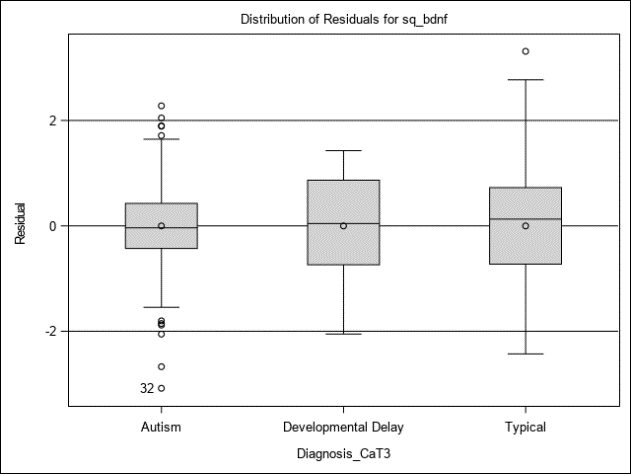


1. **Genotype distributions by group.** Summary: there were no meaningful differences in genotype distribution among groups.
   1. **Frequency table of genotype by diagnosis.** Table is presented in long format to allow for easy manipulation in a spreadsheet program. ASD = autism spectrum disorder; DD = developmental delay; TYP = typical development.

| **SNP** | **Group** | **Genotype** | **n** | **%** |
| --- | --- | --- | --- | --- |
| rs10501087 | ASD | CC | 2 | 2.13 |
| rs10501087 | ASD | TC | 25 | 26.6 |
| rs10501087 | ASD | TT | 67 | 71.28 |
| rs10501087 | DD | CC | 0 | 0 |
| rs10501087 | DD | TC | 6 | 30 |
| rs10501087 | DD | TT | 14 | 70 |
| rs10501087 | TYP | CC | 0 | 0 |
| rs10501087 | TYP | TC | 16 | 31.37 |
| rs10501087 | TYP | TT | 35 | 68.63 |
| rs11030096 | ASD | CC | 18 | 19.57 |
| rs11030096 | ASD | TC | 39 | 42.39 |
| rs11030096 | ASD | TT | 35 | 38.04 |
| rs11030096 | DD | CC | 3 | 14.29 |
| rs11030096 | DD | TC | 9 | 42.86 |
| rs11030096 | DD | TT | 9 | 42.86 |
| rs11030096 | TYP | CC | 9 | 18 |
| rs11030096 | TYP | TC | 21 | 42 |
| rs11030096 | TYP | TT | 20 | 40 |
| rs11030104 | ASD | CC | 0 | 0 |
| rs11030104 | ASD | TC | 26 | 27.66 |
| rs11030104 | ASD | TT | 68 | 72.34 |
| rs11030104 | DD | CC | 1 | 4.76 |
| rs11030104 | DD | TC | 6 | 28.57 |
| rs11030104 | DD | TT | 14 | 66.67 |
| rs11030104 | TYP | CC | 1 | 1.92 |
| rs11030104 | TYP | TC | 16 | 30.77 |
| rs11030104 | TYP | TT | 35 | 67.31 |
| rs12273539 | ASD | CC | 1 | 1.06 |
| rs12273539 | ASD | TC | 13 | 13.83 |
| rs12273539 | ASD | TT | 80 | 85.11 |
| rs12273539 | DD | CC | 1 | 4.76 |
| rs12273539 | DD | TC | 2 | 9.52 |
| rs12273539 | DD | TT | 18 | 85.71 |
| rs12273539 | TYP | CC | 1 | 1.96 |
| rs12273539 | TYP | TC | 4 | 7.84 |
| rs12273539 | TYP | TT | 46 | 90.2 |
| rs12291063 | ASD | CC | 18 | 19.57 |
| rs12291063 | ASD | TC | 39 | 42.39 |
| rs12291063 | ASD | TT | 35 | 38.04 |
| rs12291063 | DD | CC | 3 | 14.29 |
| rs12291063 | DD | TC | 9 | 42.86 |
| rs12291063 | DD | TT | 9 | 42.86 |
| rs12291063 | TYP | CC | 9 | 18 |
| rs12291063 | TYP | TC | 21 | 42 |
| rs12291063 | TYP | TT | 20 | 40 |
| rs1491850 | ASD | CC | 16 | 18.6 |
| rs1491850 | ASD | CT | 37 | 43.02 |
| rs1491850 | ASD | TT | 33 | 38.37 |
| rs1491850 | DD | CC | 4 | 20 |
| rs1491850 | DD | CT | 8 | 40 |
| rs1491850 | DD | TT | 8 | 40 |
| rs1491850 | TYP | CC | 7 | 14.89 |
| rs1491850 | TYP | CT | 25 | 53.19 |
| rs1491850 | TYP | TT | 15 | 31.91 |
| rs1491851 | ASD | CC | 20 | 23.26 |
| rs1491851 | ASD | CT | 38 | 44.19 |
| rs1491851 | ASD | TT | 28 | 32.56 |
| rs1491851 | DD | CC | 2 | 9.52 |
| rs1491851 | DD | CT | 13 | 61.9 |
| rs1491851 | DD | TT | 6 | 28.57 |
| rs1491851 | TYP | CC | 16 | 31.37 |
| rs1491851 | TYP | CT | 19 | 37.25 |
| rs1491851 | TYP | TT | 16 | 31.37 |
| rs6265 | ASD | CC | 69 | 73.4 |
| rs6265 | ASD | CT | 25 | 26.6 |
| rs6265 | ASD | TT | 0 | 0 |
| rs6265 | DD | CC | 15 | 71.43 |
| rs6265 | DD | CT | 5 | 23.81 |
| rs6265 | DD | TT | 1 | 4.76 |
| rs6265 | TYP | CC | 35 | 68.63 |
| rs6265 | TYP | CT | 15 | 29.41 |
| rs6265 | TYP | TT | 1 | 1.96 |
| rs7127507 | ASD | CC | 8 | 8.7 |
| rs7127507 | ASD | TC | 49 | 53.26 |
| rs7127507 | ASD | TT | 35 | 38.04 |
| rs7127507 | DD | CC | 4 | 20 |
| rs7127507 | DD | TC | 5 | 25 |
| rs7127507 | DD | TT | 11 | 55 |
| rs7127507 | TYP | CC | 9 | 17.65 |
| rs7127507 | TYP | TC | 21 | 41.18 |
| rs7127507 | TYP | TT | 21 | 41.18 |
| rs908867 | ASD | CC | 77 | 81.91 |
| rs908867 | ASD | CT | 17 | 18.09 |
| rs908867 | DD | CC | 17 | 80.95 |
| rs908867 | DD | CT | 4 | 19.05 |
| rs908867 | TYP | CC | 43 | 82.69 |
| rs908867 | TYP | CT | 9 | 17.31 |
| rs925946 | ASD | GG | 42 | 44.68 |
| rs925946 | ASD | GT | 45 | 47.87 |
| rs925946 | ASD | TT | 7 | 7.45 |
| rs925946 | DD | GG | 11 | 55 |
| rs925946 | DD | GT | 7 | 35 |
| rs925946 | DD | TT | 2 | 10 |
| rs925946 | TYP | GG | 24 | 46.15 |
| rs925946 | TYP | GT | 20 | 38.46 |
| rs925946 | TYP | TT | 8 | 15.38 |

- 1. **Summary of models of analysis.** Within the DD group, there was a cell size <10 even after collapsing the heterozygous and minor variants of all SNPs. Thus, they were not included in parametric analysis. For two SNPs (RS908867 and RS12273539), cell size for the TYP group remained <10 after collapsing heterozygous and minor variants, so parametric analysis was not performed. RS1491851 was analyzed without collapsing variants (the planned ordinal logistic regression; the assumption of proportional odds was supported), and the remaining eight SNPs were analyzed using binary logistic regression (major versus combined heterozygous and minor; RS1491850, RS7127507, RS12291063, RS11030104, RS6265, RS10501087, RS925946, RS11030096).

| Genotype | Collapsing of Variants by Diagnostic Group | Cell size < 10 | Analysis |
| --- | --- | --- | --- |
| RS1491851 | None | DD (Major) | Exclude DD; genotype is ordinal |
| RS1491850 | Minor into hetero | DD (Major) | Exclude DD; genotype is binary |
| RS908867 | None | DD (Hetero), TYP (Hetero), No minor variants for any group | Cannot perform analysis |
| RS7127507 | Minor into hetero | DD (Hetero + Minor) | Exclude DD; genotype is binary |
| RS12291063 | Minor into hetero | DD (Major) | Exclude DD; genotype is binary |
| RS11030104 | Minor into hetero | DD (Hetero) | Exclude DD; genotype is binary |
| RS12273539 | Minor into hetero, TYP combined = 5 | DD (Hetero + Minor), TYP (Hetero + Minor) | Cannot perform analysis |
| RS6265 | Minor into hetero | DD (Hetero + Minor) | Exclude DD; genotype is binary |
| RS10501087 | Minor into hetero | DD (Hetero) | Exclude DD; genotype is binary |
| RS925946 | Minor into hetero | DD (Hetero + Minor) | Exclude DD; genotype is binary |
| RS11030096 | Minor into hetero | DD (All) | Exclude DD; genotype is binary |

proc logistic data=data;

where group NE ‘DD’;

class genotype group (ref='ASD') / param=reference;

model genotype(descending) = group ;

run;

- 1. **Group comparison of genotype distribution**. Odds ratios (ASD / TYP) predicting minor allele; odds ratios > 1 indicate that the odds of the minor allele were greater for the ASD group than for the TYP. ASD = autism spectrum disorder; TYP = typical development; OR = odds ratio; CI = confidence interval.

| Genotype | Odds Ratio | OR 95% CI Lower Limit | OR 95% CI Upper Limit | Wald Chi-Square | Wald Chi-Square p |
| --- | --- | --- | --- | --- | --- |
| RS10501087 | 0.88 | 0.42 | 1.85 | 0.11 | 0.74 |
| RS11030096 | 1.09 | 0.54 | 2.20 | 0.05 | 0.82 |
| RS11030104 | 1.27 | 0.61 | 2.65 | 0.41 | 0.52 |
| RS12291063 | 1.09 | 0.54 | 2.20 | 0.05 | 0.82 |
| RS1491850 | 0.75 | 0.36 | 1.60 | 0.55 | 0.46 |
| RS1491851 | 0.80 | 0.42 | 1.52 | 0.46 | 0.50 |
| RS6265 | 1.26 | 0.60 | 2.67 | 0.37 | 0.54 |
| RS7127507 | 1.14 | 0.57 | 2.29 | 0.14 | 0.71 |
| RS925946 | 0.94 | 0.48 | 1.86 | 0.03 | 0.86 |

1. **Results of models comparing BDNF concentration among genotypes, controlling for platelet count.** General syntax is shown; model was run iteratively for all SNPs. (a) The identity of the SNP for each model is found in the “Model Information” section, under “Group Effect”. Four SNPs had small cell size and the minor genotype was collapsed into the heterozygous genotype for analysis. (b) Parameter estimate (B) of the change in square-root transformed BDNF for a one-unit increase in number of minor alleles (major genotype = 0, heterogeneous genotype = 1, minor genotype = 2) is summarized for all SNPs.

| SNP | Collapse for analysis |
| --- | --- |
| RS1491851 | None |
| RS1491850 | None |
| RS908867 | None |
| RS7127507 | None |
| RS12291063 | None |
| RS11030104 | Minor into hetero |
| RS12273539 | Minor into hetero |
| RS6265 | Minor into hetero |
| RS10501087 | Minor into hetero |
| RS925946 | None |
| RS11030096 | None |

proc mixed data=data plots=all;

class genotype;

model bdnf_sqrt = platelet genotype_linear / s;

repeated / group= genotype;

lsmeans genotype / pdiff cl;

run;

1. **Full results of each model.**

| **Model Information** | |
| --- | --- |
| **Data Set** | WORK.BDNF3 |
| **Dependent Variable** | sq_bdnf |
| **Covariance Structure** | Variance Components |
| **Group Effect** | RS10501087_c |
| **Estimation Method** | REML |
| **Residual Variance Method** | None |
| **Fixed Effects SE Method** | Model-Based |
| **Degrees of Freedom Method** | Between-Within |

| **Solution for Fixed Effects** | | | | | |
| --- | --- | --- | --- | --- | --- |
| **Effect** | **Estimate** | **Standard Error** | **DF** | **t Value** | **Pr > \|t\|** |
| **Intercept** | 2.6504 | 0.4444 | 155 | 5.96 | <.0001 |
| **Platelet** | 0.003397 | 0.001150 | 155 | 2.95 | 0.0036 |
| **RS10501087_c_l** | 0.1731 | 0.1737 | 155 | 1.00 | 0.3204 |

| ***The Mixed Procedure*** |
| --- |

| **Model Information** | |
| --- | --- |
| **Data Set** | WORK.BDNF3 |
| **Dependent Variable** | sq_bdnf |
| **Covariance Structure** | Variance Components |
| **Group Effect** | rs11030096 |
| **Estimation Method** | REML |
| **Residual Variance Method** | None |
| **Fixed Effects SE Method** | Model-Based |
| **Degrees of Freedom Method** | Between-Within |

| **Solution for Fixed Effects** | | | | | |
| --- | --- | --- | --- | --- | --- |
| **Effect** | **Estimate** | **Standard Error** | **DF** | **t Value** | **Pr > \|t\|** |
| **Intercept** | 3.2645 | 0.4483 | 153 | 7.28 | <.0001 |
| **Platelet** | 0.003690 | 0.001166 | 153 | 3.17 | 0.0019 |
| **rs11030096_l** | -0.2793 | 0.1252 | 153 | -2.23 | 0.0272 |

| ***The Mixed Procedure*** |
| --- |

| **Model Information** | |
| --- | --- |
| **Data Set** | WORK.BDNF3 |
| **Dependent Variable** | sq_bdnf |
| **Covariance Structure** | Variance Components |
| **Group Effect** | rs11030104_c |
| **Estimation Method** | REML |
| **Residual Variance Method** | None |
| **Fixed Effects SE Method** | Model-Based |
| **Degrees of Freedom Method** | Between-Within |

| **Solution for Fixed Effects** | | | | | |
| --- | --- | --- | --- | --- | --- |
| **Effect** | **Estimate** | **Standard Error** | **DF** | **t Value** | **Pr > \|t\|** |
| **Intercept** | 2.6649 | 0.4467 | 157 | 5.97 | <.0001 |
| **Platelet** | 0.003396 | 0.001162 | 157 | 2.92 | 0.0040 |
| **rs11030104_c_l** | 0.1459 | 0.1737 | 157 | 0.84 | 0.4023 |

| ***The Mixed Procedure*** |
| --- |

| **Model Information** | |
| --- | --- |
| **Data Set** | WORK.BDNF3 |
| **Dependent Variable** | sq_bdnf |
| **Covariance Structure** | Variance Components |
| **Group Effect** | RS12273539_c |
| **Estimation Method** | REML |
| **Residual Variance Method** | None |
| **Fixed Effects SE Method** | Model-Based |
| **Degrees of Freedom Method** | Between-Within |

| **Solution for Fixed Effects** | | | | | |
| --- | --- | --- | --- | --- | --- |
| **Effect** | **Estimate** | **Standard Error** | **DF** | **t Value** | **Pr > \|t\|** |
| **Intercept** | 2.1137 | 0.4635 | 156 | 4.56 | <.0001 |
| **Platelet** | 0.004201 | 0.001173 | 156 | 3.58 | 0.0005 |
| **RS12273539_c_l** | 0.4264 | 0.2038 | 156 | 2.09 | 0.0381 |

| ***The Mixed Procedure*** |
| --- |

| **Model Information** | |
| --- | --- |
| **Data Set** | WORK.BDNF3 |
| **Dependent Variable** | sq_bdnf |
| **Covariance Structure** | Variance Components |
| **Group Effect** | rs12291063 |
| **Estimation Method** | REML |
| **Residual Variance Method** | None |
| **Fixed Effects SE Method** | Model-Based |
| **Degrees of Freedom Method** | Between-Within |

| **Solution for Fixed Effects** | | | | | |
| --- | --- | --- | --- | --- | --- |
| **Effect** | **Estimate** | **Standard Error** | **DF** | **t Value** | **Pr > \|t\|** |
| **Intercept** | 3.2721 | 0.4492 | 153 | 7.28 | <.0001 |
| **Platelet** | 0.003666 | 0.001169 | 153 | 3.14 | 0.0021 |
| **rs12291063_l** | -0.2771 | 0.1252 | 153 | -2.21 | 0.0284 |

| ***The Mixed Procedure*** |
| --- |

| **Model Information** | |
| --- | --- |
| **Data Set** | WORK.BDNF3 |
| **Dependent Variable** | sq_bdnf |
| **Covariance Structure** | Variance Components |
| **Group Effect** | rs1491850 |
| **Estimation Method** | REML |
| **Residual Variance Method** | None |
| **Fixed Effects SE Method** | Model-Based |
| **Degrees of Freedom Method** | Between-Within |

| **Solution for Fixed Effects** | | | | | |
| --- | --- | --- | --- | --- | --- |
| **Effect** | **Estimate** | **Standard Error** | **DF** | **t Value** | **Pr > \|t\|** |
| **Intercept** | 2.8959 | 0.4387 | 144 | 6.60 | <.0001 |
| **Platelet** | 0.003036 | 0.001217 | 144 | 2.50 | 0.0137 |
| **rs1491850_l** | 0.04112 | 0.1145 | 144 | 0.36 | 0.7201 |

| ***The Mixed Procedure*** |
| --- |

| **Model Information** | |
| --- | --- |
| **Data Set** | WORK.BDNF3 |
| **Dependent Variable** | sq_bdnf |
| **Covariance Structure** | Variance Components |
| **Group Effect** | rs1491851 |
| **Estimation Method** | REML |
| **Residual Variance Method** | None |
| **Fixed Effects SE Method** | Model-Based |
| **Degrees of Freedom Method** | Between-Within |

| **Solution for Fixed Effects** | | | | | |
| --- | --- | --- | --- | --- | --- |
| **Effect** | **Estimate** | **Standard Error** | **DF** | **t Value** | **Pr > \|t\|** |
| **Intercept** | 2.7488 | 0.4583 | 148 | 6.00 | <.0001 |
| **Platelet** | 0.003260 | 0.001171 | 148 | 2.78 | 0.0061 |
| **rs1491851_l** | 0.04129 | 0.1195 | 148 | 0.35 | 0.7303 |

| ***The Mixed Procedure*** |
| --- |

| **Model Information** | |
| --- | --- |
| **Data Set** | WORK.BDNF3 |
| **Dependent Variable** | sq_bdnf |
| **Covariance Structure** | Variance Components |
| **Group Effect** | RS6265_c |
| **Estimation Method** | REML |
| **Residual Variance Method** | None |
| **Fixed Effects SE Method** | Model-Based |
| **Degrees of Freedom Method** | Between-Within |

| **Solution for Fixed Effects** | | | | | |
| --- | --- | --- | --- | --- | --- |
| **Effect** | **Estimate** | **Standard Error** | **DF** | **t Value** | **Pr > \|t\|** |
| **Intercept** | 2.6752 | 0.4508 | 156 | 5.93 | <.0001 |
| **Platelet** | 0.003511 | 0.001180 | 156 | 2.98 | 0.0034 |
| **RS6265_c_l** | 0.1100 | 0.1793 | 156 | 0.61 | 0.5406 |

| ***The Mixed Procedure*** |
| --- |

| **Model Information** | |
| --- | --- |
| **Data Set** | WORK.BDNF3 |
| **Dependent Variable** | sq_bdnf |
| **Covariance Structure** | Variance Components |
| **Group Effect** | rs7127507 |
| **Estimation Method** | REML |
| **Residual Variance Method** | None |
| **Fixed Effects SE Method** | Model-Based |
| **Degrees of Freedom Method** | Between-Within |

| **Solution for Fixed Effects** | | | | | |
| --- | --- | --- | --- | --- | --- |
| **Effect** | **Estimate** | **Standard Error** | **DF** | **t Value** | **Pr > \|t\|** |
| **Intercept** | 2.5437 | 0.4415 | 153 | 5.76 | <.0001 |
| **Platelet** | 0.003764 | 0.001197 | 153 | 3.15 | 0.0020 |
| **rs7127507_l** | 0.1216 | 0.1406 | 153 | 0.86 | 0.3884 |

| ***The Mixed Procedure*** |
| --- |

| **Model Information** | |
| --- | --- |
| **Data Set** | WORK.BDNF3 |
| **Dependent Variable** | sq_bdnf |
| **Covariance Structure** | Variance Components |
| **Group Effect** | rs908867 |
| **Estimation Method** | REML |
| **Residual Variance Method** | None |
| **Fixed Effects SE Method** | Model-Based |
| **Degrees of Freedom Method** | Between-Within |

| **Solution for Fixed Effects** | | | | | |
| --- | --- | --- | --- | --- | --- |
| **Effect** | **Estimate** | **Standard Error** | **DF** | **t Value** | **Pr > \|t\|** |
| **Intercept** | 2.1097 | 0.4789 | 157 | 4.40 | <.0001 |
| **Platelet** | 0.004022 | 0.001183 | 157 | 3.40 | 0.0009 |
| **rs908867_l** | 0.4627 | 0.2239 | 157 | 2.07 | 0.0404 |

| ***The Mixed Procedure*** |
| --- |

| **Model Information** | |
| --- | --- |
| **Data Set** | WORK.BDNF3 |
| **Dependent Variable** | sq_bdnf |
| **Covariance Structure** | Variance Components |
| **Group Effect** | rs925946 |
| **Estimation Method** | REML |
| **Residual Variance Method** | None |
| **Fixed Effects SE Method** | Model-Based |
| **Degrees of Freedom Method** | Between-Within |

| **Solution for Fixed Effects** | | | | | |
| --- | --- | --- | --- | --- | --- |
| **Effect** | **Estimate** | **Standard Error** | **DF** | **t Value** | **Pr > \|t\|** |
| **Intercept** | 2.5498 | 0.4321 | 156 | 5.90 | <.0001 |
| **Platelet** | 0.003808 | 0.001188 | 156 | 3.21 | 0.0016 |
| **rs925946_l** | 0.1064 | 0.1407 | 156 | 0.76 | 0.4506 |

1. **Summary of the effect of genotype on estimated square-root transformed BDNF concentration**. Estimate is the slope, or the estimated change in square-root transformed BDNF for a one-unit change in number of minor alleles.

| SNP | Estimate | Standard Error | Estimate 95% CI Lower | Estimate 95% CI Upper | DF | t Value | p |
| --- | --- | --- | --- | --- | --- | --- | --- |
| RS10501087 | 0.17 | 0.17 | -0.17 | 0.52 | 155 | 1 | 0.3204 |
| RS11030096 | -0.28 | 0.13 | -0.53 | -0.03 | 153 | -2.23 | 0.0272 |
| RS11030104 | 0.15 | 0.17 | -0.20 | 0.49 | 157 | 0.84 | 0.4023 |
| RS12273539 | 0.43 | 0.20 | 0.02 | 0.83 | 156 | 2.09 | 0.0381 |
| RS12291063 | -0.28 | 0.13 | -0.52 | -0.03 | 153 | -2.21 | 0.0284 |
| RS1491850 | 0.04 | 0.11 | -0.19 | 0.27 | 144 | 0.36 | 0.7201 |
| RS1491851 | 0.04 | 0.12 | -0.19 | 0.28 | 148 | 0.35 | 0.7303 |
| RS6265 | 0.11 | 0.18 | -0.24 | 0.46 | 156 | 0.61 | 0.5406 |
| RS7127507 | 0.12 | 0.14 | -0.16 | 0.40 | 153 | 0.86 | 0.3884 |
| RS908867 | 0.46 | 0.22 | 0.02 | 0.91 | 157 | 2.07 | 0.0404 |
| RS925946 | 0.11 | 0.14 | -0.17 | 0.38 | 156 | 0.76 | 0.4506 |
